# Multigenerational inequalities of opportunity in health outcomes

**DOI:** 10.1186/s12939-024-02144-0

**Published:** 2024-07-10

**Authors:** Namal N. Balasooriya, Jayatilleke S. Bandara, Nicholas Rohde

**Affiliations:** 1 Centre for Health Services Research, Faculty of Medicine, University of Queensland, UQ Health Sciences Building, RBWH Campus Central, Fig Tree Dr, Herston, Brisbane, QLD 4006 Australia; 2 Department of Accounting, Finance and Economics, Griffith University, Brisbane, Australia; 3 Department of Accounting, Finance and Economics, Griffith University, Gold Coast, Australia

**Keywords:** D63, I12, Multigenerational inequality, Inequality of opportunity, Health outcomes, Social class, Health behaviours, Children’s health

## Abstract

This paper studies multigenerational health transmission mechanisms in Australian panel data. Using inequality-of-opportunity (IOP) models, we demonstrate that grandparental socioeconomic status (SES) is an important determinant of personal health, even after controlling for health and SES at the parental level. Our findings hold over a range of health/biomarkers of individuals’ physical and mental well-being and appear to be especially sensitive to educational outcomes on the father’s side. Since ingrained socioeconomic (dis)advantages that persist over multiple generations may be indicative of social class, our results suggest that subtle attitudinal and behavioural characteristics associated with this variable may be a key factor driving health disparities.

**Supplementary Information:**

The online version contains supplementary material available at 10.1186/s12939-024-02144-0.

## Introduction

Economic inequalities are often passed down from generation to generation, where parents’ socioeconomic status (SES) influences their children’s distribution of outcomes. For example, children from wealthier or more-educated parents are known to be healthier [5, 26, 48], achieve better educational outcomes [73], and do better in labour markets [32]. Correlations between parental characteristics and child outcomes are often interpreted under the umbrella of inequality of opportunity (IOP), which are harmful disparities that lie beyond personal control.

In this paper, we apply IOP concepts to Australian health data, but we extend the standard econometric models to consider the effects that individuals’ grandparents may play in this process. We model the impacts of grandparental SES on individuals’ health while also controlling for analogous parental traits. This allows our models to capture the direct effect of grandparental status rather than the effect that flows through the intermediate (i.e., parental) generation. That is, we consider the idea that health status is not just driven by parental characteristics but by ingrained socioeconomic disparities apparent over multiple generations.

Why might grandparental SES be a source of unequal opportunity in health, even once the effects of parental characteristics are removed? We suggest two key mechanisms that may produce this type of result. On the one hand, attitudes and behaviours related to health might be passed down when grandparents have close contact with grandchildren [11, 69]. In this case, grandparental caregiving may contribute directly to children’s health. Family backgrounds (e.g., ethnicity, residential arrangements, number of children, and parental and grandparental SES) may also matter for grandparental caregiving practices (see [80]).

On the other hand, economic, social, and cultural factors associated with social class, as proxied by the length of time a family has held a given level of social status, may also matter. Advantages and disadvantages shift from one generation to the next, but the effects also depend on the duration that individuals experience life within a specific social class [17, 31, 33, 44, 72, 82]. For instance, different sets of cultural values exist for families that hold high levels of social status for several generations [17, 44, 82], and these values may influence a variety of health and social behaviours.

Our analysis used six health-related markers for physical and mental well-being that identify significant effects of grandparental SES in all cases. The estimates are most decisive for body mass index (BMI), mental health, and physical health variables, although the breadth of the results across indicators is a key finding. We also found that the effect transmits mostly through grandpaternal rather than grandmaternal lines. Since fathers (and grandfathers) tend to perform breadwinning roles, while mothers and grandmothers are more likely to play caring roles, our correlations suggest that the socioeconomics associated with material well-being is likely to be especially important.

To quantify the proportional impacts of different types of variables, we used regression-based econometric decompositions (i.e., Owen values) with health outcomes as the dependent variable. These results show that grandparental characteristics explain a similar proportion of IOP in Australian health compared to parental characteristics. For our physical health indicators, the explained contribution of grandparents’ SES to their grandchildren’s health ranged from 8 to 29% of explained inequality, while the corresponding figures for our parental SES variables were from 8 to 23%.^1^ The surprisingly high proportion of inequality attributed to grandparental characteristics suggests that there may be substantial omitted-variable problems associated with the standard two-generation model used to study inequality in health.

Our work ties a broader literature on inequality with respect to predetermined SES. The central idea here is that background characteristics or circumstances reflect factors that lie beyond personal control and are, therefore, a source of unfair inequality. This is distinct from efforts—factors that individuals have control over and, therefore, lie within the domain of personal responsibility [75]. The existing research on IOP in health [1, 2, 8, 12, 14, 27, 52, 78, 79, 85] have considered parental characteristics as proxies for circumstances. However, we are unaware of any study that has measured IOP in health by considering the effect of the grandparents–grandchild relationship on health inequality.

The rest of the paper is structured as follows. The following section, Methodology section, describes the data source, variables, and sample characteristics. Data section examines the role of the multigenerational association in IOP in health by applying regression models demonstrating that grandparental SES is a significant determinant of IOP. Health outcomes section decomposes total predetermined inequality into contributions from different sets of circumstances, and Explanatory variables section discusses some important results. The final section, Summary statistic section, presents a summary and conclusion.

## Methodology

### Data

Our data consists of 17 waves (from 2001 to 2017) of the Household Income and Labour Dynamics in Australia (HILDA) Survey. HILDA is a large, nationally representative, random sample of more than 17,000 individuals from more than 7,000 Australian households. Since 2001, HILDA has collected data for individuals’ health status, demographic, and socioeconomic backgrounds using face-to-face interviews and self-completion questionaries.

To conduct our analysis, we required a multigenerational dataset assembled from HILDA by matching observations across three consecutive generations. Children (i.e., the first generation) are matched to their parents using cross-wave identifiers assigned separately for both mother and father. We could then link grandparental characteristics with corresponding grandchildren because parents respond to their parents’ SES questions. Our sample limits individuals aged 15 years or above, while the requirement of having data on parents and grandparents restricted our sample to mostly young individuals (basic characteristics of our subsample are outlined further below).

### Health outcomes

We employ six different characteristics of health as outcome variables. The first two are biological indicators of body composition. BMI is one of our outcomes of interest because body mass has been considered as one of the risk factors for non-communicable diseases. BMI is a ratio of weight in kilograms over the square of height in metres and is calculated in HILDA using self-reported mass and height. Using BMI as a health measure has some standard caveats. It is not sensitive to the deferent between body fat and muscle and does not consider the types of fat, which have a different metabolic effects, and which parts of the body contain more fat. However, BMI has been a widely used health measure in health-related research, showing a robust association with non-communicable diseases [70, 83]. We also define another variable to measure overweightness (BMI*), using the following formula to tackle non-monotonicities between bodyweight and health (see [4]).

1 $${BMI}^\ast=\left\{\begin{array}{l}0\;if\;BMI\;\leq25\\BMI-25\;if\;BMI\;>\;25\end{array}\right.$$

Four other measures are sourced as indicators of individuals’ mental health (MH), physical health (PH), general health (GH), and health satisfaction (HS). MH, PH, and GH outcomes are measured using the SF-36 questionnaire, which is a widely employed health assessment tool [87]. Each aggregated health measure uses 36 questions to form a scale ranging from 0 to 100, where higher values indicate healthier outcomes. Lastly, we take data on self-reported HS, which is a standard subjective marker, where respondents can select a number between ‘0’ (no satisfaction in health) and ‘10’ (highest satisfaction in health).

### Explanatory variables

Our main explanatory variables are sets of grandparental SES measures (both from the mother’s side and the father’s side). These include markers of education, occupation, whether the grandparents are divorced, and several other indicators of economic conditions. The level of education is measured using the level of schooling completion of both the grandfathers and grandmothers. Grandparents’ occupational prestige is captured with a scale variable ranging from ‘0’ to ‘100’ and is measured according to the Australian and New Zealand standard classifications [65]. The other grandparental economic conditions are recorded with dummies indicating whether the grandfather was unemployed for at least six months while the parents were growing up and whether grandfathers or grandmothers were in paid employment when parents were aged 14 years.

Following the standard IOP model, which regresses individuals’ outcomes against their parents’ characteristics, we take a series of variables related to parental economic attainments. The set of parental SES includes the level of schooling completion, occupation, whether the parents are divorced, whether the father was unemployed for at least six months when the respondent was growing up, and whether the mother or father was in paid employment when the respondent was aged 14 years. The measurements of these variables are similar to those for grandparental SES.

We also include measures of parental health because these outcomes may also be transmitted across generations by non-socioeconomic channels, e.g., each individual inherits a unique set of genes from both paternal and maternal lines [53]. Also, these factors may capture the indirect effect of family economic uncertainty on children’s health, e.g., transmission via poor mental health and unhealthy behaviours [22, 23, 55]. Parental health status is proxied by both the mothers’ and fathers’ BMI, MH, PH, GH aggregates, and HS. Scales of these parental health variables are the same as the measures of children’s health outcomes. We also control some family background markers and demographic variables: age, gender, ethnicity, living area, country of birth, and first language.

### Summary statistics

Table 1 summarises the distribution of all outcome variables and independent variables at baseline. In our study sample, most of the respondents are young (Figure A1 in the appendix shows more than 98% of respondents are aged between 15 and 30 years). As shown in Table 1, the average age of individuals in the sample was approximately 18 years, whereas the respondents’ maximum age was 59 years. Therefore, our work measured multigenerational socioeconomic disparities in health inequalities in younger individuals. As a result, our inequality estimates are likely to be relatively low as health disparities are known to increase over the lifespan [38].

**Table 1:** Descriptive statistics

Variable	Mean	Std. dev	Min	Max
**Demographic characteristics**				
Age	18.486	4.276	11	59
Female	0.476	0.499	0	1
Language: English	0.984	0.124	0	1
Refugee	0.003	0.057	0	1
Indigenous origin	0.014	0.117	0	1
Country of birth: Australia	0.963	0.189	0	1
**Health outcomes**				
BMI	23.205	4.365	12.8	50.9
Distance from healthy BMI	1.131	2.624	0	25.9
Satisfaction: health condition	8.233	1.512	0	10
Mental health	73.938	16.443	4	100
General health	75.076	18.233	0	100
Physical health	93.746	17.005	0	100
**Grandparents SES: father’s side**				
Grandfather schooling: not respond	0.986	0.116	0	1
Primary and secondary	0.007	0.082	0	1
Year 11 and year 12	0.007	0.083	0	1
Grandmother schooling: not respond	0.986	0.116	0	1
Primary and secondary	0.004	0.065	0	1
Year 11 and year 12	0.009	0.096	0	1
Grandfather in paid employment	0.038	0.191	0	1
Grandmother in paid employment	0.624	0.484	0	1
Grandparent divorced	0.076	0.265	0	1
Grandfather unemployed	0.073	0.261	0	1
Grandfather’s occupation	42.796	21.314	0	100
Grandmother’s occupation	39.993	23.076	3.4	100
**Grandparental SES: mother’s side**				
Grandfather in paid employment	0.002	0.046	0	1
Grandmother in paid employment	0.610	0.488	0	1
Grandparent divorced	0.114	0.318	0	1
Grandfather unemployed	0.097	0.296	0	1
Grandfather’s occupation	46.891	23.036	7.9	100
Grandmother’s occupation	41.232	21.902	3.4	100
**Parental SES**				
Father schooling: no response	0.001	0.034	0	1
Non	0.251	0.433	0	1
Primary and secondary	0.439	0.496	0	1
Year 11 and year 12	0.309	0.462	0	1
Mother schooling: no response	0.168	0.374	0	1
Primary and secondary	0.520	0.500	0	1
Year 11 and year 12	0.312	0.463	0	1
Father in paid employment	0.915	0.278	0	1
Mother in paid employment	0.835	0.371	0	1
Parents divorced	0.011	0.104	0	1
Father unemployed	0.115	0.320	0	1
Father’s occupation	53.131	24.058	4.9	100
Mother’s occupation	54.452	22.802	3.4	100
**Parental health**				
BMI mother	27.512	5.946	16.3	58.2
Mother satisfaction: health condition	7.457	1.540	0	10
Mother physical health	88.082	16.397	0	100
Mother general health	72.798	18.413	0	100
Mother mental health	76.230	15.254	0	100
BMI (father)	28.082	4.409	18.2	49.4
Father satisfaction: health condition	7.316	1.556	0	10
Father physical health	86.850	19.510	0	100
Father general health	68.313	18.217	0	100
Father mental health	77.142	15.534	0	100
Age (mother)	48.387	5.803	30	83
Age(father)	50.618	6.241	32	89

*Note*: In this table, the second column presents the variables’ average values over the estimated sample, which includes 5215 respondents. The standard deviation, minimum value, and maximum value of each variable are presented from the third column to the fifth column, respectively. For binary variables, mean values refer to sample proportion with given characteristics

Aside from age distribution, considering the gender ratio, our sample is relatively representative of the Australian population. Our observations include an almost equal proportion of females (47.6%) and males (52.4%). However, most respondents’ born language is English (98%) and were born domestically (96%), while approximately 1% of our sample are Indigenous and 0.3% are refugees.

## A multigenerational IOP model

To measure IOP in health, we employed the standard parametric approach, which uses regression models to attribute variations in outcomes to a set of predetermined circumstance variables (e.g., see [13, 19, 36, 76]. Thus, the overall variation in the health indicator represents the total inequality, while the explained component represents the inequality due to unequal opportunity. The unexplained (residual) term represents unobserved circumstances and individual efforts. Other demographic variables are sometimes used as controls.

A baseline IOP model for health outcome ${y}_{it}$ is given below. This baseline model additively partitions inequality into contributions from parental SES (first sigma term), parental health status (second sigma term), demographics (third sigma term), and an unexplained component (captured by ${u}_{it}$). The ordinary least squares regression estimates of EQ (2) presents in Table A1 in the appendix.

2 $$y_{it}=a_0+\sum_{p=1}^qy_{1p}PSES_{pit}+\sum_{r=1}^sy_{2r}\;PHS_{rit}\;+\;\sum_{v=1}^wy_{3v}D_{vit}\;+\;u_{it}$$

Our augmented model appears below. As in the baseline model, ${\mathrm{\alpha }}_{0}$ is an intercept and ${u}_{it}$ is the error term. Variable ${y}_{it}$ represents each of our six health outcomes (*BMI*_*it,*_* BMI*_it_^*^_,_ *MH*_*it*_*, PH*_*it*_*, GH*_*it,*_ and *SH*_*it*_) of individual $i$ in time *t*;${PGP}_{j}$, ${\forall }_{j}\in \left(1\dots k\right)$ and${MGP}_{l}$, ${\forall }_{l}\in \left(1\dots m\right)$ represent paternal grandparents’ SES and maternal grandparents’ SES, respectively.${PSES}_{p} {,\forall }_{p}\in \left(1\dots q\right)$,${PHS}_{r}$,${\forall }_{r}\in \left(1\dots s\right)$,${D}_{v}$, ${\forall }_{v}\in \left(1\dots w\right)$ represent the control variables: parental SES, parental health status, and individuals’ demographic factors and family background*.*${\beta }_{1}$,${\beta }_{2 }, {\gamma }_{1}$,${\gamma }_{2}$, and ${\gamma }_{3}$ are parameter vectors to be estimated.

3 $$y_{it}=a_0+\;\sum_{j=1}^k\beta_{1j}PGP_{jit}+\;\sum_{l=1}^m\beta_{2l}MGP_{lit}+\sum_{p=1}^qy_{1p}PSES_{pit}+\sum_{r=1}^sy_{2r}\;PHS_{rit}+\sum_{v=1}^wy_{3v}D_{vit}+u_{it}$$

The model specified in EQ (3) is fitted to our multigenerational dataset and the results are reported in Table 2.

**Table 2:** Effect of circumstances on individual health: regressions coefficients

	BMI	BMI*	HS	MH	GH	PH
**Grandparental SES: father’s side**						
Grandfather education	0.124***	-0.038	-0.022	-0.148	-0.345*	-0.039
Grandmother education	-0.243***	-0.045	0.071***	0.192	0.637***	0.104
Grandfather in paid employment	0.436	0.244	-0.128	2.129	1.402	3.673***
Grandmother in paid employment	-0.211	-0.178	0.036	-1.625*	-0.381	0.094
Grandparent divorced	0.239	0.344	0.156	0.181	3.101	0.599
Grandfather unemployed	0.789	0.695	-0.552***	-3.680**	-7.513***	-0.905
Grandfather’s occupation	-0.014**	-0.005	0.004*	-0.02	-0.02	0.005
Grandmother’s occupation	0.013**	0.003	-0.001	-0.001	-0.004	0.005
**Grandparental SES: mother’s side**						
Grandfather in paid employment	2.386	0.962	0.309	0.223	8.047*	-12.418
Grandmother in paid employment	-0.058	-0.01	-0.131*	-0.891	-0.945	0.53
Grandparent divorced	0.141	0.037	0.154	-0.061	1.434	-1.934
Grandfather unemployed	0.051	-0.229	0.195*	2.660**	3.597**	2.127
Grandfather’s occupation	-0.004	-0.003	0.003	0	0.023	0.032*
Grandmother’s occupation	0.014**	0.004	-0.001	0.028	-0.027	-0.044*
**Parental SES**						
^(a)^Father schooling: non	4.036***	1.149**	0.346	-1.824	-6.566*	-3.609
Primary and secondary	3.735***	1.005*	0.274	-0.379	-6.373**	-2.217
Year 11 and year 12	4.497***	1.217	0.125	-5.863	-10.829**	-7.474**
^(a)^Mother schooling: primary and secondary	0.549	0.244	-0.185*	-1.743	-2.730*	-1.621
Year 11 and year 12	0.015	0.227	-0.11	0.372	0.197	4.034*
Father in paid employment	0.821	0.272	-0.082	-1.706	-0.626	-1.09
Mother in paid employment	-0.373	-0.269	-0.126	-1.829	-1.003	-0.062
Parents divorced	2.033*	0.856	-0.696*	-6.153	-8.489	-3.069
Father unemployed	-0.676	-0.332	-0.096	-3.236**	-1.896	-2.758
Father’s occupation	-0.004	-0.005	0	-0.016	0.015	0.016
Mother’s occupation	-0.013*	-0.007	0.002	0.013	0.008	0.02
**Parental health**						
BMI mother	0.165***	0.072***	-0.001	-0.048	-0.038	-0.09
Mother satisfaction: health condition	0.03	0.009	-0.011	-0.153	-0.543*	0.450*
Mother physical health	-0.008	-0.012**	0.002	0.019	0.009	0.002
Mother general health	0.006	0.006	0.008***	0.045	0.115***	-0.026
Mother mental health	-0.002	-0.001	0.009***	0.196***	0.170***	0.043
BMI (father)	0.261***	0.135***	-0.011	0.117	-0.105	-0.066
Father satisfaction: health condition	0.106	0.059	0.064***	0.603**	0.314	0.427
Father physical health	-0.007	-0.001	0	0.007	0.002	0.003
Father general health	0.007	-0.001	-0.002	-0.006	0.056*	-0.012
Father mental health	-0.004	0	0	0.060**	-0.032	-0.018
Age (mother)	0.099**	0.056**	-0.012	-0.337**	-0.143	0.072
Age(father)	-0.086**	-0.064**	0.013	0.144	-0.039	-0.102
**Demographic factors**						
Age	0.596***	0.235***	-0.128***	0.041	-0.06	0.411
Age2	-0.008***	-0.003***	0.001	-0.004	-0.001	-0.005
Female	-0.111	0.065	-0.372***	-3.917***	-4.424***	-0.51
Refugee	-2.494	-1.292*	-0.91	-12.962	-0.72	2.368
Indigenous origin	-0.794	-0.936*	-0.548*	-14.700**	-13.222**	-5.407
Area of living	0.33	0.264*	-0.009	0.402	-0.532	-1.654*
Born in Australia	-1.193	-0.806	-0.348	0.534	0.974	2.427
English	2.090*	1.13	-0.262	-0.285	-3.962	-5.433**
Constant	-2.326	-8.009***	9.312***	63.593***	84.256***	94.453***
R-squared	0.266	0.191	0.124	0.125	0.107	0.035
N	4928	4928	5215	5108	5108	5099

This table presents regression coefficients of covariates in EQ (3), which considers six different health outcomes: BMI, BMI*, HS, MH, PH, and GH. We estimated those models using ordinary least squares and used heteroskedasticity-robust standard errors throughout. Reference categories are male, non-refugee, non-indigenous, born in out of Australia, the language start of speak is not English and not respond (a)

* p < 0.10, ** p < 0.05, *** p < 0.01

Once the models were fitted, we used the variance of outcomes as our inequality metric. This measure is proportional to the squared coefficient of variation, which is a member of the additively decomposable index below.

4 $${I}_{\alpha }\left(y\right)=\frac{1}{N\alpha (\alpha -1)}\sum_{i=1}^{N}\left[{\left(\frac{{y}_{i}}{\widehat{y}}\right)}^{\alpha }-1\right]$$

Here ${I}_{\alpha }\left(y\right)$ is the health inequality index and $\alpha$ a weighting parameter, which sets the index equal to half the squared coefficient of variation when $\alpha =2$. We can model the fraction of total inequality explained by our model covariates using the ratio ${I}_{\alpha }\left(\widehat{y}\right)/{I}_{\alpha }\left(y\right)$, The advantage of this measure is that this ratio is equal to the ${R}^{2}$ term from a regression model used to estimate $\widehat{y}$ (i.e., the fitted values from EQ [2] and EQ [3]).

We can, therefore, use the coefficient of determination terms reported in Table 2 to identify the overall fractions of inequality captured by our parameters. The results show that our models explain 3–26.6% of inequality in our health makers. The highest values belong to the BMI and overweightness measures, while the general physical health variable had an explained component of only 3%. Also, around 10–12.5% of variation of the SF-36 indicators of MH, GH and measure of healthy life satisfaction is captured by our models.

The results from Table 2 indicate that grandparental SES is an important predictive variable across a spectrum of health outcomes. Alongside traditional determinants, such as parental health and education, grandparental educational and employment status variables are significant in a number of regressions. This is especially true for grandfathers, which suggests an economic (rather than caregiving) channel may be responsible. In addition, there are estimates of offsetting signs for grandmothers and grandfathers in some instances, which is consistent with collinearity between grandparents’ SESs (see Table A2). For this reason, we recommend focusing on the aggregate effect rather than individual covariates.

## The contribution of grandparents to inequality of opportunity in health

In order to boil-down aggregate contributions from each set of covariates in EQ (3), we employed Owen’s (1977) decomposition of the R-squared term. This econometric approach is a relative of the Shapley value decomposition [18, 81] and is useful for dealing with clusters of related variables within a single model. In our case, the Owen index improved the decomposition because it satisfied several important theoretical properties, including the symmetric treatment of variable subgroups [54]. Moreover, considering these properties of Owen value decomposition, [49] suggested that the Owen index is most suitable if at least a subgroup has more than one exogenous variable.

In EQ (3), we employed 42 (K) exogenous variables divided into four subgroups (G) representing grandparental SES, parents’ SES, parental health status, and demographic characteristics. A permutation $\pi$ is compatible concerning G if variables in each group arrange in the permutation contiguously. So, the Owen index (*OW*_*j*_*)* for calculating the decomposition of total explained inequality (R^2^) in health outcomes $\left({H}_{j}^{m},\forall m\in \left(1\dots \dots .m\right)\right)$ is given by

5 $$H_j^m={OW}_j\left(K,R^2,G\right)=\frac1{\left|\Pi\left(K.G\right)\right|}{\textstyle\sum_{\pi\in\Pi\left(K,G\right)}}R^2\left(P_j^\pi\right)-R^2\left(P_j^\pi\backslash\left\{j\right\}\right)$$

Here, ${R}^{2}\left({P}_{j}^{\pi }\right)- {R}^{2}\left({P}_{j}^{\pi }\backslash \left\{j\right\}\right)$ is a marginal contribution of *j’s* variable when variables appear as $\pi \in \Pi \left(K,G\right)$ in the model. Table 3 presents the calculated decomposition of predetermined inequality in health by groups of circumstances.

**Table 3:** Decomposition of explained inequality in health

Health outcome	Source of predetermined inequality	Contribution (%)	Confidence interval (95%)	
			Lower	Upper
BMI	Grandparents SES	8.542	6.119	11.466
	Parents SES	10.519	8.278	13.186
	Parents health	60.590	55.599	65.444
	Demographic factors	20.220	16.388	23.989
BMI*	Grandparents SES	10.298	6.602	15.453
	Parents SES	13.698	10.916	17.353
	Parents health	57.532	50.695	64.013
	Demographic factors	18.072	13.678	23.769
HS	Grandparents SES	16.930	11.905	22.264
	Parents SES	10.922	7.281	15.731
	Parents health	34.557	28.577	41.207
	Demographic factors	37.276	30.454	43.433
MH	Grandparents SES	8.957	5.410	12.960
	Parents SES	14.982	10.878	21.206
	Parents health	50.136	42.596	57.997
	Demographic factors	25.479	19.345	32.800
PH	Grandparents SES	26.437	17.016	38.136
	Parents SES	28.091	17.688	37.845
	Parents health	23.996	15.014	34.708
	Demographic factors	20.281	11.407	32.262
GH	Grandparents SES	18.831	13.564	24.616
	Parents SES	11.065	6.742	16.116
	Parents health	47.564	40.541	54.756
	Demographic factors	22.275	16.786	28.376

*Note*: Column three in this table presents the percentage of each source of circumstances’ contribution to the total predetermined inequality (R-squared), obtained from 500 bootstrapped samples with a 95% significant level. For the decomposition analysis, we considered the six regression estimates of our health outcomes. These models are the same as the models presented in Table 2. Refer to Table 2 for the R-squared value of each estimated model

Although our regression estimates (see Table 2) indicate a small impact of grandparental SES on health, decomposition calculation highlights that ignoring this grandparental effect may lead to underestimating the IOP measure. The decomposition results in Table 3 show that grandparental SES is an equally (or more) important factor of health inequality as the effect of parents’ SES. For example, grandparental SES contributes 8–26% to IOP in health, while the contribution of parental SES ranges from 8–23%. Moreover, grandparental SES and parental SES are responsible for 10% and 13% of IOP in MH outcomes, respectively.

Considering parental health status, the decomposition results in Table 3 show that the contribution to IOP in health is significantly higher than other circumstances sources. It ranges between approximately 31% and 62% across the different health outcomes. This result is plausible because, on the one hand, health transmission across the generation mostly occurs via genetic interaction [84]. On the other hand, a considerable amount of health inequality is explained by genetic traits [24, 66]. The demographic factors and other family background markers account for approximately 20–34% of the total IOP in health.

## Discussion

Given that we found that grandparental factors account for a substantial fraction of predetermined inequality in health, there is some value in identifying plausible causal mechanisms that account for this result. Here we return to the two potential explanations outlined in the introduction: (a) the effects of grandparental caregiving and (b) the potential effects of cultural attitudes associated with social class.

### Caregiving

Correlations between grandparental SES and child health may appear if higher-status grandparents offer better care compared to lower-status grandparents. To create the patterns observed in our data, such an effect needs to be direct and not operate via an intermediate channel, such as parental behaviour. However, considerable evidence exists suggesting that such a direct effect exists. For example, grandparental caregiving is known to positively impact their grandchildren’s survival, physical growth, and injury protection [59, 77]. Grandparents can also provide informal medical advice and health-related economic resources that do not flow through parents, e.g., by diagnosing illnesses [45] and by providing money for doctors and treatments [46]. Evidence also suggests that the influence of grandparental caregiving can be negative because of promoting unhealthy food habits and behaviours [74, 89]. The potential for negative effects to be transmitted through social channels, e.g., intrafamilial conflict (between parents and grandparents), also exists and may be a source of stress and lead to diminished health.

To be able to explain our results, these tendencies need to be stronger (in a positive or beneficial sense) for grandparents with higher educational attainments. Such a link is highly plausible, e.g., higher SES grandparents are less likely to be absent; therefore, they have a greater capacity to play a caring role [60]. And education is usually a predictor of better health behaviours [25], which would likely spill over into better caring skills.

### Cultural factors

Our second proposed explanation is that our results might reflect the latent effects of social class. The key idea here is that families that have held a higher status for longer may take on cultural attitudes that are middle or upper-class, while families that have been poorer for multiple generations may exhibit working-class cultural norms. This may be because either individuals’ childhood SES positively associates with a level of education and employment [61] or lifestyle behaviours and attitudes are transmitted through multiple generations [6, 16, 40]. Notably, the class effect is separate from the parental SES effect because ingrained cultural attitudes are distinct from purely economic variables, such as income. Thus, some children may grow up in a relatively affluent environment with cultural attitudes reminiscent of working-class families, while others may be exposed to cultural norms that are predicted by their parents’ incomes or education levels [41, 42, 62]

The social class’s objective resources (such as educational achievements, occupational prestige, and wealth) create cultural identities among the working class and middle- or upper-social-class individuals [56]. From childhood, individuals in different social classes have experienced different material life cultures: different neighbourhoods and peers, belonging to different educational institutions and social clubs, engaging with different recreational events, eating different food, and enjoying fashion with different brands. Individuals from affluent cultural backgrounds also have greater opportunities to make choices, stand up for their rights, and live in a secure environment where they can acquire basic needs [62].

These cultural differences between the working class and middle- or upper-social class individuals may feed through to health behaviours. If decisions to smoke [9, 71], binge drink [86], overeat [50], or engage in substance abuse [57] are informed by culture, then we would expect to see a greater associated health problems in these segments of the population [28, 61]. Other potential causal channels, such as psychological stress or depression due to childhood status [68], job uncertainty [88], working environment [58], or violent behaviour [43], may also be present.

The transmission of stress, mainly through allostatic load, may significantly shape health outcomes [64]. Disadvantaged populations, particularly those exposed to excessive psychosocial and environmental stressors, are at increased risk of developing chronic diseases, contributing to health disparities [7]. Moreover, these effects can be transmitted across generations. For instance, prenatal experiences can influence stress axis function, predisposing individuals to various pathologies and transgenerational memory of fatal experiences extendsacross multiple generations [63].

### Peer effects

Grandparents may also affect their grandchildren’s health by influencing neighbourhood and peer effects. Here, the causal mechanism is that grandparents may provide additional resources that affect the social groups of their grandchildren, which then go on to affect behaviours. For example, high SES grandparents may shape their grandchildren’s peer groups by influencing educational choices through their social, cultural, and economic capital [67]. Moreover, since grandparental SES influences educational and employment success [3, 29], relationships built up through individuals’ SES may be partially influenced by childhood inherited background.

The distinction between neighbourhoods and peer exposure indicates that social-class culture during childhood generates subtle attitudinal and behavioural characteristics associated with individual health outcomes [21, 34]. Differences in behaviours are also associated with peer behaviour, e.g., adolescent smoking is motivated by their friends’ smoking behaviour [47], and children’s food intake is influenced by their friends’ food intake [37]. Likewise, some important aspects of the neighbourhood, such as access to common built facilities (recreational or leisure parks, physical activity establishments [15], neighbourhood socioeconomic background [51], and neighbourhood unsafe environments [39], can be responsible for the variation of childhood experiences that drive individuals’ health behaviours, attitudes, and psychological traits.

### Limitations

Although including grandparents’ factors in the IOP model marks a notable enhancement, since we use explained variation by the variables chosen in the model (circumstances) as a measure for IOP, there is a chance to omit some portion of observed IOP, such as the factors beyond Social class. Nevertheless, omitted circumstances might be associated with the circumstance variables included in the model [10, 35]. That means IOP due to unobserved circumstances is partly accounted for in the IOP derived from the empirical model. Therefore, we define the estimation derived by the multigenerational IOP model as lower boundaries of actual IOP.

Also, the influence of grandparents on health is conditional on the quality and consistency of the relationships among generations [80]. This relationship may be disrupted by various factors, such as a grandparent’s or parent’s death, geographical distance, or involvement in the justice or child protection systems [20, 30]. However, our data set limits analysing the persistence of the relationship among generations since the parental and grandparental SES are time-invariant in our data set.

## Conclusion

This paper has studied multigenerational IOP in health using Australian data. We showed that across a spectrum of MH and PH outcomes, markers of grandparental SES predict their grandchildren’s health outcomes, even after controlling for parents’ equivalent socioeconomic characteristics. We then performed some econometric decompositions attributing explained inequalities to various clusters of variables related to intergenerational inequalities. Our results are surprising because they place approximately equal weights on the contributions of parents and grandparents, which suggests that more complicated causal flows are present beyond those implicit in standard intergenerational inequality models.

We speculate on two possible explanations for this result. First, grandparents may play an important role in caregiving, and more educated grandparents may do a better job raising healthier children. For example, these grandparents may be more likely to be present within the family (and add to the stock of caring resources available) or be better suited to identifying or treating health complaints. They may also provide financial resources to their grandchildren that do not flow directly through the parents. Second, we argue that grandparents of higher SES may generate different cultural attitudes in ways reminiscent of social class. These attitudes may feed through to affect a variety of behaviours, such as tobacco or alcohol consumption.

Our results have some general implications for the measurement of IOP. IOP models, which are typically lower-bound estimates because socioeconomic constraints are only partially observable, commonly produce estimates that seem too low. Our decompositions indicate that neglecting multigenerational factors may explain some of this missing inequality.

We suggest prioritising investments in early life, particularly in maternal and child health, education, and support systems that target disadvantaged families to break the cycle of inequality. Also, broader structural and social changes are important, including combatting systemic discrimination and ensuring equal access to education, employment, and healthcare.

### Supplementary Information

**Supplementary Material 1.**

## Data Availability

The unit record data from the HILDA Survey were obtained from the Australian Data Archive, which is hosted by The Australian National University.

## Footnotes

1 Although we have used a similar set of SES variables for both parents and grandparents, we considered grandparents from the father and mother sides. However, we have not included grandparents' education for the maternal side due to the multicollinearity issue.
