# Supplementary material for: Multigenerational inequalities of opportunity in health outcomes
Source: Int J Equity Health. 2024 Jul 10;23:140. doi: 10.1186/s12939-024-02144-0 (PMC11234677; doi:10.1186/s12939-024-02144-0)
Supplement: Supplementary file 1 — Supplementary Material 1. [file 12939_2024_2144_MOESM1_ESM.docx]

**Appendix**

**Figure A1**

***Distributions Age and Outcome Variables – HILDA Sample***

Note: The figure presents the histograms of two sets of variables. The first part shows the age distributions of respondents and their parents. The second part presents the distributions of outcome variables. Data from the estimation sample (n=5215) has been used throughout.

Table A1 Regression Results of Standard IOP model

|  | BMI | BMI* | HS | MH | GH | PH |
| --- | --- | --- | --- | --- | --- | --- |
| **Parental SES** |  |  |  |  |  |  |
| (a)Father schooling: non | 3.962*** | 0.931** | 0.378** | -2.91 | -8.301*** | -6.097*** |
| Primary and secondary | 3.663*** | 0.790** | 0.314** | -1.568 | -8.074*** | -4.375*** |
| Year 11 and year 12 | 4.622*** | 1.085 | 0.136 | -6.835** | -12.482*** | -9.758*** |
| (a)Mother schooling: primary and secondary | 0.534 | 0.217 | -0.185* | -1.919 | -2.829** | -1.539 |
| Year 11 and year 12 | -0.168 | 0.155 | -0.094 | 0.149 | 0.036 | 4.026* |
| Father in paid employment | 0.793 | 0.259 | -0.069 | -1.622 | -0.671 | -1.175 |
| Mother in paid employment | -0.376 | -0.296 | -0.107 | -2.263* | -0.964 | -0.005 |
| Parents divorced | 2.103* | 0.926 | -0.749* | -6.316 | -8.172 | -2.711 |
| Father unemployed | -0.559 | -0.281 | -0.116 | -3.017* | -1.548 | -2.292 |
| Father’s occupation | -0.005 | -0.006 | 0 | -0.017 | 0.008 | 0.015 |
| Mother’s occupation | -0.009 | -0.006 | 0.002 | 0.019 | 0.012 | 0.017 |
| **Parental health** |  |  |  |  |  |  |
| BMI mother | 0.170*** | 0.075*** | -0.003 | -0.041 | -0.037 | -0.097 |
| Mother satisfaction: health condition | 0.031 | 0.006 | -0.001 | -0.065 | -0.399 | 0.417 |
| Mother physical health | -0.011 | -0.014*** | 0.003 | 0.028 | 0.023 | 0.012 |
| Mother general health | 0.007 | 0.008* | 0.007*** | 0.036 | 0.103*** | -0.029 |
| Mother mental health | -0.002 | -0.001 | 0.009*** | 0.186*** | 0.163*** | 0.043 |
| BMI (father) | 0.260*** | 0.134*** | -0.008 | 0.117 | -0.092 | -0.079 |
| Father satisfaction: health condition | 0.102 | 0.058 | 0.059** | 0.565* | 0.234 | 0.372 |
| Father physical health | -0.008 | -0.001 | 0.001 | 0.009 | 0.01 | 0.005 |
| Father general health | 0.009 | -0.001 | -0.001 | -0.004 | 0.054* | -0.013 |
| Father mental health | -0.008 | -0.003 | 0.001 | 0.064** | -0.015 | -0.004 |
| Age (mother) | 0.096** | 0.051* | -0.009 | -0.307** | -0.1 | 0.089 |
| Age(father) | -0.087** | -0.061** | 0.009 | 0.144 | -0.069 | -0.126 |
| Demographic factors |  |  |  |  |  |  |
| Age | 0.591*** | 0.230*** | -0.129*** | 0.013 | -0.059 | 0.412 |
| Age2 | -0.008*** | -0.003*** | 0.001* | -0.004 | -0.002 | -0.005 |
| Female | -0.096 | 0.088 | -0.385*** | -3.949*** | -4.532*** | -0.615 |
| Refugee | -2.683 | -1.649** | -0.592 | -10.613 | 2.43 | 2.731 |
| Indigenous origin | -0.955 | -0.889* | -0.536* | -14.142** | -12.887** | -5.916 |
| Area of living | 0.365 | 0.268* | -0.04 | 0.616 | -0.464 | -1.529* |
| Born in Australia | -1.111 | -0.847 | -0.335 | 1.16 | 1.662 | 0.933 |
| English | 1.848* | 1.115 | -0.172 | 0.313 | -2.416 | -4.140** |
| Constant | -1.439 | -7.593*** | 9.093*** | 60.677*** | 79.463*** | 97.412*** |
| R-squared | 0.249 | 0.18 | 0.106 | 0.115 | 0.088 | 0.027 |
| N | 4928 | 4928 | 5215 | 5108 | 5108 | 5099 |
| Note: * p<0.10, ** p<0.05, ***p<0.01. This table presents regression coefficients of covariates in EQ (2), which considers six different health outcomes that are BMI, BMI*, satisfaction about health (HS), mental health (MH), general health (GH), and physical health (PH). We estimated those models using ordinary least squares and use heteroskedasticity-robust standard errors throughout. Reference categories are male, non-refugee, non-indigenous, born in out of Australia, the language start of speak is not English and not respond (a) | | | | | | |

Table A2: Correlation Matrix: Grandparental Characteristics

| Father’s side | | | | | | | | |
| --- | --- | --- | --- | --- | --- | --- | --- | --- |
|  | GFE | GME | GFPE | GMPE | GPD | GFU | GFO | GMO |
| GFE | 1 |  |  |  |  |  |  |  |
| GME | 0.613 | 1 |  |  |  |  |  |  |
| GFPE | 0.018 | 0.091 | 1 |  |  |  |  |  |
| GMPE | -0.003 | 0.010 | -0.016 | 1 |  |  |  |  |
| GPD | -0.003 | -0.008 | -0.048 | 0.082 | 1 |  |  |  |
| GFU | -0.001 | 0.009 | 0.020 | -0.019 | 0.109 | 1 |  |  |
| GFO | 0.013 | -0.002 | -0.003 | -0.078 | 0.022 | -0.046 | 1 |  |
| GMO | 0.017 | 0.032 | 0.008 | 0.030 | 0.032 | 0.017 | 0.359 | 1 |
| Mother’s side | | | | | | | | |
| GFE | 1 |  |  |  |  |  |  |  |
| GME | 0.105 | 1 |  |  |  |  |  |  |
| GFPE | 0.022 | 0.505 | 1 |  |  |  |  |  |
| GMPE | 0.024 | 0.006 | 0.018 | 1 |  |  |  |  |
| GPD | 0.011 | 0.012 | -0.016 | 0.061 | 1 |  |  |  |
| GFU | 0.009 | 0.002 | -0.016 | -0.039 | 0.072 | 1 |  |  |
| GFO | 0.002 | -0.018 | -0.012 | -0.018 | -0.003 | -0.088 | 1 |  |
| GMO | 0.009 | -0.004 | 0.008 | 0.063 | -0.026 | -0.084 | 0.392 | 1 |
| Note: This table presents the correlation coefficients between grandparental characteristics. Abbreviations: Grandfather education (GFE), Grandmother education (GME), Grandfather in paid employment (GFPE), Grandmother in paid employment (GMPE), Grandparent divorced (GPD), Grandfather unemployed (GFU), Grandfather’s occupation (GFO) and Grandmother’s occupation (GMO). | | | | | | | | |
